# A Curious Case of Gastrointestinal Eosinophilia Induced by Treatment With Immune Checkpoint Inhibitors

**DOI:** 10.14309/crj.0000000000001075

**Published:** 2023-06-14

**Authors:** Yala Kirthi Reddy, Inderbitzin Sonya Fischer, Joanna Kolodney, Megan Willard

**Affiliations:** 1 Department of Gastroenterology, West Virginia University, Morgantown, WV; 2 Department of Hematology and Oncology, West Virginia University, Morgantown, WV

**Keywords:** immune check point inhibitors, immunotherapy, gastrointestinal eosinophilia

## Abstract

Immune checkpoint inhibitors have revolutionized oncologic treatment. However, they are linked to various side effects,^1^ a rare one being gastrointestinal eosinophilia. We present a patient with malignant melanoma treated with nivolumab. She underwent upper endoscopy 6 months later which showed a duodenal ulcer and linear furrows of her esophagus. Biopsies of the esophagus, stomach, and duodenum were consistent with eosinophilic infiltration. Repeat endoscopy after nivolumab discontinuation revealed near-complete resolution of eosinophilia in the stomach and duodenum, with lingering eosinophilia in the esophagus. The purpose of this report was to increase awareness of gastrointestinal eosinophilia associated with checkpoint inhibitors.

## INTRODUCTION

The use of immune checkpoint inhibitors (ICIs) in several malignancies has significantly improved treatment outcomes. Unfortunately, ICI therapy is associated with specific immune-related adverse events (IRAEs). IRAEs can affect a wide range of organs with varied presentations, severity, and durations.^2^ Hypereosinophilia associated with checkpoint inhibitors is rarely reported. Eosinophil counts can increase to levels where hypereosinophilic visceral complications can occur.^3^ One such presentation of visceral hypereosinophilia IRAE is gastrointestinal eosinophilia. Primary eosinophilic gastrointestinal disorder is a rare disorder defined by eosinophilic infiltration in the wall of the gastrointestinal tract in the absence of other established causes of tissue eosinophilia.^4^ There are data to suggest that eosinophilia may persist for many months after discontinuation of ICIs.^5^

## CASE REPORT

A 40-year-old White woman had a 1-year history of superficial spreading malignant melanoma (stage IIIA pT1b pN1a cM0) of the upper back. Two months after diagnosis, the patient underwent wide local excision with right axillary sentinel lymph node biopsy. She then started adjuvant nivolumab 480 mg every 4 weeks for a duration of 12 cycles. Nivolumab, a fully human IgG4 monoclonal antibody against PD-1 receptors, blocks the interaction of PD-1 on the T cell and PD-L1/PD-L2 on the tumor cell improving the antitumor function of the T cells. The US Food and Drug Administration has approved nivolumab for the treatment of several malignancies, including malignant melanoma.^3^

**Figure 1.**
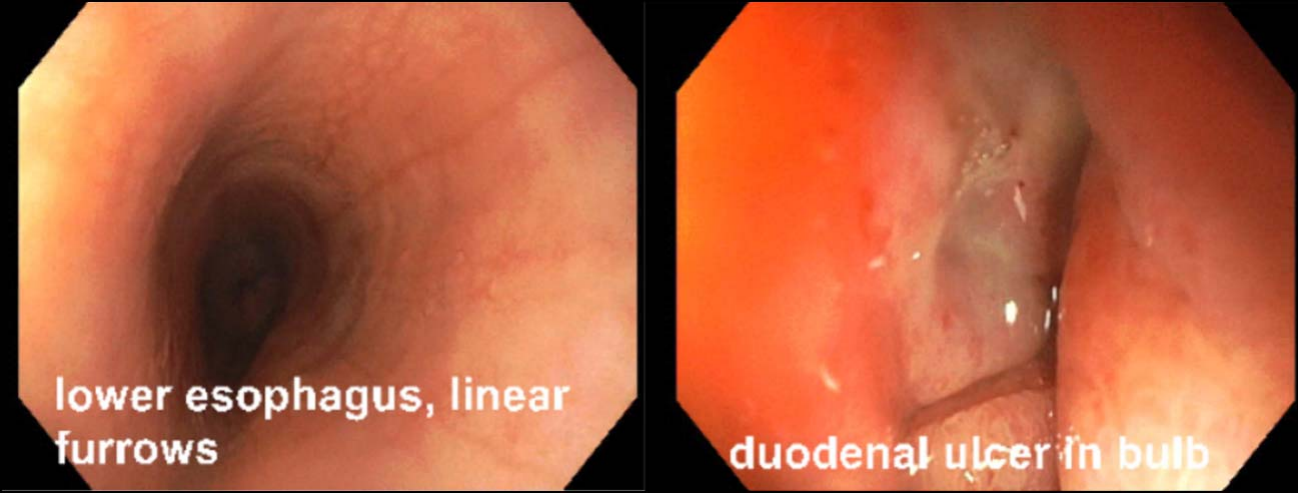
Images from patient's first upper endoscopy. Mild linear furrows noted in the mid and distal esophagus (left). Clean based ulcer was seen in the duodenal bulb (right).

The patient was referred to gastroenterology for nonspecific abdominal pain. She underwent an upper endoscopy with biopsy around 6 months after initiation of immunotherapy. The endoscope showed mild linear furrows in the mid and lower esophagus. The stomach mucosa was normal. A 1 cm nonbleeding cratered ulcer was found in the duodenal bulb (Figure 1). Small intestinal and stomach biopsies showed many eosinophils. The distal and proximal esophagus revealed numerous intraepithelial eosinophils (up to 363 and 298 per high-power field, respectively) and many eosinophilic micro abscesses.

On further questioning, the patient reported that she had a history of seasonal allergies dating back to childhood. She had an upper endoscopy for dysphagia 3 years before her diagnosis of melanoma, which revealed stenosis in her gastroesophageal junction. Biopsies at the time were consistent with mild reflux esophagitis and no evidence of eosinophilia.

**Figure 2.**
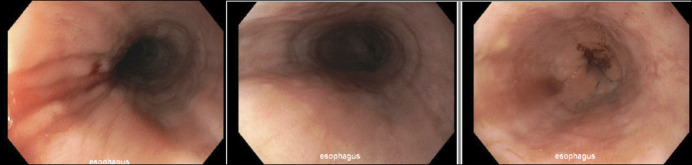
Images from the patient’s second upper endoscopy, which showed mild linear furrows in the esophagus.

Based on the patient's overall clinical history and biopsy results, it was believed that the patient had gastrointestinal eosinophilia secondary to immunotherapy. Given the mild symptoms, it was decided to not stop nivolumab. She was not started on steroids and did not make any significant dietary changes. She was instead started on a proton pump inhibitor and sucralfate to aid in healing of the duodenal ulcer. Eight months later, the patient reported that she was feeling well and denied dysphagia, abdominal pain, and heart burn. She had a repeat upper endoscopy 6 months after completing adjuvant nivolumab, which revealed that the duodenal ulcer had resolved but linear furrows persisted in the esophagus (Figure 2). Biopsies revealed persistent eosinophilia in her esophagus >40 per high powered field. Duodenum and stomach biopsies showed no residual eosinophilia. It should be noted throughout her evaluations that her hemogram and differential remained within normal ranges and without peripheral eosinophilia. She never developed any symptoms concerning for involvement of other organ systems, and her gastrointestinal symptoms never progressed after the initiation of therapy.

## DISCUSSION

ICIs have revolutionized the treatment of various cancers. However, ICIs have been associated with a diverse range of IRAEs. The most common systems where IRAEs occur are the lungs, endocrine system, skin, gastrointestinal tract, and musculoskeletal systems.^6^ However, a new class of IRAEs called hematological IRAEs (heme-IRAEs) are rare and potentially severe.^5^ A study by Delanoy et al discussed patients treated with anti-PD-1 or anti-PD-L1 monoclonal antibodies. Only 3.6% had heme-IRAEs, and neutropenia, autoimmune hemolytic anemia, and immune thrombocytopenia were most common.^7^ In other studies, an increase in eosinophil count has been rarely explored as a correlative marker with immunotherapy.^5^ In this study, we present a patient with gastrointestinal eosinophilia induced by nivolumab administration for malignant melanoma.

Eosinophilic enteritis is a rare primary eosinophilic gastrointestinal disorder, first described in 1937, characterized by gastrointestinal symptoms in the presence of pathological eosinophilic infiltration of the intestinal wall without secondary causes of gastrointestinal eosinophilia.^8^ Hypereosinophilia was observed in 30% of patients who were treated with ipilimumab for malignant melanoma. Seventy-seven percent of them had associated IRAEs, involving mostly the gastrointestinal tract in the form of diarrhea and colitis.^9^

While the exact mechanism for eosinophilia as a side effect of ICIs is unknown, small studies have indicated that patients treated with certain ICIs experiencing IRAEs appear to present with a diversification of the T-cell repertoire.^10,11^ It is postulated that ICIs expand tissue-reactive T-cell clones in certain patients, thus developing corresponding toxicities.^10^ Research is currently underway to identify routine blood tests, such as peripheral blood lymphocyte count, which may be predictive of response and toxicity to ICIs.^12^

Research is currently underway to predict potential IRAEs.^12^ Patients with baseline sarcopenia and low muscle attenuation are more likely to experience severe treatment-related toxicity when treated with ICIs.^13^ Several other potential baseline risk factors of severe IRAEs have also been proposed, including family history of autoimmune diseases; tumor infiltration and location; previous viral infections such as human immunodeficiency virus or hepatitis; and the concomitant use of medicines with known autoimmune toxicities such as antiarrhythmics, antibiotics, anticonvulsants, or antipsychotics.^14,15^

To our knowledge, this is one of the few reports of nivolumab-induced gastrointestinal eosinophilia. This case highlights the growing presence of eosinophilic disorders. Clinicians should evaluate for eosinophilic gastrointestinal disorders in any patient on ICIs who presents with abdominal pain, nausea, vomiting, or diarrhea. However, given the ICIs' efficacy in the treatment of various malignancies, it is suggested that asymptomatic blood eosinophilia and mild-to-moderate organ damage do not necessarily constitute sufficient grounds for treatment discontinuation.^5^ Further research related to eosinophilia and its causes in the context of varying histologies and clinical profiles of patients is warranted.

## DISCLOSURES

Author contributions: All authors contributed to this manuscript and agree to be accountable for the final version. Yala Kirthi Reddy is the article guarantor.

Financial disclosure: None to report.

Informed consent was obtained for this case report.
